# The Influence of Maximum Squatting Strength on Jump and Sprint Performance: A Cross-Sectional Analysis of 492 Youth Soccer Players

**DOI:** 10.3390/ijerph19105835

**Published:** 2022-05-11

**Authors:** Michael Keiner, Torsten Brauner, Björn Kadlubowski, Andre Sander, Klaus Wirth

**Affiliations:** 1 Department of Exercise Sciences, German University of Health & Sport, 85737 Ismaning, Germany; bjoernkadlubowski@gmail.com; 2 Department of Kinesiology & Biomechanics, German University of Health & Sport, 85737 Ismaning, Germany; torsten.brauner@dhgs-hochschule.de; 3 DSC Arminia Bielefeld e. V., 33615 Bielefeld, Germany; 4 German Luge and Bobsled Federation, 83471 Berchtesgaden, Germany; a.sander@bsd-portal.de; 5 Department of Sports Science, University of Applied Sciences Wiener Neustadt, 2700 Vienna, Austria; klaus.wirth@fhwn.ac.at

**Keywords:** 1RM, squat jump, counter movement jump, linear sprint

## Abstract

This study aims to analyze the influence of relative strength performance, determined by parallel back squats (REL SQ), on 30 m sprinting (LS) and on jumping performance (squat [SJ], countermovement [CMJ]) in a large sample (*n* = 492) of elite youth soccer players. The soccer players were divided into subgroups based on their strength performance: strength level 1 (0.0–0.5 REL SQ), strength level 2 (>0.5–1.0 REL SQ), strength level 3 (>1.0 to 1.5 REL SQ), strength level 4 (>1.5 to 2.0 REL SQ), and strength level 5 (>2.0 REL SQ). The results of this study show that REL SQ explains 45–53% (r = |0.67–0.73|) of the variance of SJ, CMJ, and LS for the total sample. Strength levels 2–4 showed similar coefficients of correlation in jumping performance (r = |0.42–0.55|) and strength levels 2 and 3 in sprint performance (r = |0.41|). The respective extreme strength levels showed lower coefficients of correlation with the sprinting and jumping performance variables (r = |0.11–0.29|). No coefficients could be calculated for strength level 5 because no athlete achieved an appropriate strength level (>2.0 REL SQ). The data from this study show a clear influence of REL SQ on sprint and jump performance, even in a large sample.

## 1. Introduction

Competitive soccer requires a complex mix of technical, tactical, and conditional skills [1,2]. During the 90 min of a game, athletes cover distances of over 10 km [3,4,5,6,7]. Even though an athlete performs on average only 15.5 jumps per game [8], and sprinting (<30 m) [9] only accounts for 0.5–3.0% of effective playing time [4,10,11,12] and 1–11% of the distance covered [3,4,6], these speed–strength actions, according to detailed analyses, seem to determine the result of the game [3,13,14,15,16]. Therefore, to be successful, it is mandatory for soccer players to develop high-level jumping and sprinting performances [1,16].

Strength plays a key role in jumping and sprinting performance, as athletes need to exert great forces within short time intervals during push-off and landing. Reported peak values of vertical ground reaction forces during push-off are up to and sometimes even more than twice of the athletes’ body weight [17] and, during landings, range between four and six times their body weight [18]. During the start of a sprint, ground reaction forces have also been reported to be two to five times the athlete’s body weight [19,20] and, even by the end of the positive acceleration phase, still manifest between two and three times their body weight [21,22,23]. To prepare athletes adequately, increasing maximum strength, in particular high-velocity strength, and power are of primary concern in monitoring and training soccer players. Despite the need to generate strength at high velocities, maximum strength itself seems to play a major role, as it has been shown that athletes with high maximum strength levels are most likely the athletes with the highest values of maximum power output [24,25,26,27,28].

Maximum strength, as a basic strength ability, positively influences the performance of explosive strength [29]. Several studies have analyzed the relationships between maximum strength and sprint or jump performance in soccer [30,31,32,33,34,35,36], utilizing various methods including isometrics (r = |0.23–0.80|) [30,34,37,38], isokinetics (r = |0.22–0.48|) [34], and free-weight squats (r = |0.28–0.94|) [30,31,32,34,36]. The findings in previous studies suggest an influence of absolute (r = |0.02–0.94|) [30,32,34,36,39,40,41,42,43] and relative strength (maximum strength performance divided by body weight [REL], r = |0.01–0.72|) [30,32,42,43] on various sprinting and jumping performances. These results, however, are based on small samples (*n* = 15–34) of trained soccer players of varying ages (16.8 ± 0.6–25.1 ± 4.56) and differ in the performed squat technique (e.g., half vs. deep). The differences in correlation coefficients between these studies might be due to sample bias and, consequently, fluctuate around the true correlation coefficient of the entire population of soccer players. Another possible reason for the discrepancies between studies may be found in the different strength levels of the analyzed players. According to Suchomel’s theory, the relationship between back squat performance and speed–strength capability, such as in sprinting or jumping, is not solely linear but rather S-shaped [44]. Suchomel [44] categorizes the strength capability REL into three different strength levels for the squat performance (SQ): 0–0.5 REL SQ, 0.5–2.0 REL SQ, and >2.0 REL SQ. The lowest strength level is limited by the individual’s motor learning ability, and consequently underestimates actual strength performance. At the other end of the scale, with athletes showing strength capabilities greater than 2.0 REL SQ, the transfer of strength into the movement seems to be problematic. If that is the case, studies with small sample sizes that include athletes from a broad range of back squat performances would lead to low linear correlation scores.

Therefore, the study aimed to analyze the influence of relative strength performance, determined by parallel back squats, on sprinting and jumping performance in a large sample of elite youth soccer players. It is hypothesized that relative strength correlates with jumping and sprint performance, respectively, and that this correlation differs in various administered samples defined by relative strength performance.

## 2. Materials and Methods

To answer this research question, a cross-sectional study was conducted. At the beginning of the season (July–August), 492 male youth soccer players from six youth elite training centers were evaluated in their performances in squat jump (SJ), countermovement jump (CMJ), 30 m linear sprint (LS), and 1 repetition maximum (1RM) in SQ to calculate the relationships between strength and explosive strength performances. Tests were carried out on 3 test days within a 1-week period. LS was tested on day 1, SJ and CMJ were tested on day 2, and 1RM was tested on day 3. One week prior to test day 1, the soccer players completed a familiarization session for all tests on two separate days. The elite players were familiar with the test battery because these tests were part of their semi-annual performance diagnostics routine. Data were collected between 2014 and 2021 at six youth training centers. The analyzed teams were between under 14 years old (U14) and U23. Only two U14 teams were analyzed as part of this study since only players with a proper introduction to and familiarization with the squat technique could be included safely [39,45].

### 2.1. Subjects

A total of 492 male youth soccer players were analyzed (Table 1). All teams played in the highest German youth league according to age, except for the players in the U23 category, who already competed in senior competitions at the national level. The soccer teams were classified as elite in reference to the definition used by Lorenz et al. [46], who consider elite athletes as those who play at a higher level than peers within a sport. The players regularly performed 3 to 5 (U14: 3, U15: 3–4, U17-U23: 4–5) soccer sessions per week (1.5–2 h training/day). All teams regularly competed in their respective leagues on weekends during the season. The training volume did not deviate significantly from these values during the COVID-19 pandemic (2020–2021). The subjects did not participate in fatiguing training sessions for a minimum of 2 days prior to testing. None of the subjects reported any injuries at the time of testing. Due to organizational reasons, nine soccer players missed the jump measurement and twenty-two the sprint measurement.

Each subject and their parents (if the subject was younger than 18 years) were informed about the aims of the study and the experimental risks involved with the research, and provided written informed consent. Furthermore, this study was performed in accordance with the Helsinki Declaration and was approved by the Universities Ethics Committee.

### 2.2. Procedures

The warm-up for the jump and sprint tests consisted of nonspecific running at low-to-medium intensity for approximately 5 min (min). Then, coordination exercises, such as running with lifted knees, heeling, and side stepping, were performed for approximately 5 min. Subsequently, 3 acceleration runs over approximately 30 m were performed with short intervening walking breaks.

LS performance was measured in 3 sprint attempts over a distance of 30 m each, with a 3 min break between sprints. The duration of the LS tests was measured using a double-timing gate system (wk7 time watch, Ditzingen, Germany). The starting point was marked 0.75 m away from the starting gate to avoid early triggering, e.g., by a hand movement or a bent body position. The subjects independently chose when the measurement began. Thus, reaction time was excluded from the measurement. The test–retest reliability is reported to have an average ICC = 0.90 for LS [47].

Jumping performance was measured using a contact mat (Refitronic, Schmitten, Germany) that operated as a switch. This system sent information to the computer regarding whether the mat was loaded. From this information, the flight time and the jump height were determined for all jumps. The jump height was calculated from the flight time (gt²/8; with g being the gravitational acceleration (9.81 m·s^−2^) and t = flight time in seconds). The squat jump was initiated at a knee angle of 90° without counter movement. The subjects had 5 trials for each jump in which to achieve their best result. Between every jump, the athletes received a 1 min break. The test–retest reliability is reported with ICC = 0.97 [48].

Testing included the determination of the 1RM for a back squat (high bar). The barbell was positioned on the musculus trapezius pars descendens below the seventh cervical vertebra. The participants stood erect with a self-selected width of the feet, flexed their knees and hips to reach a deep squat position with proper form (top of thigh breaking parallel) and returned to the starting position. Attempts failed when the trained spotter visually detected rounding of the back or insufficient squat depth. The determination of the 1RM was achieved with a maximum of 5 trials, after 2 submaximal sets with 6 repetitions. The rest duration between attempts was at least 5 min. The test–retest reliability of 1RM for the squat is reported between ICC = 0.91–0.99 [49]. Absolute maximum strength values were divided by body weight to determine relative strength performance (REL SQ) (REL SQ = 1RM/body mass). In addition to the classification of strength from Suchomel [44] and based on various studies evaluating the parallel squat [50,51,52,53,54,55,56,57], strength levels for the soccer players in this study were administered: strength level 1 (0.0–0.5 REL SQ), strength level 2 (>0.5–1.0 REL SQ), strength level 3 (>1.0–1.5 REL SQ), strength level 4 (>1.5–2.0 REL SQ) and strength level 5 (>2.0 REL SQ). This assessment was based on studies that have collected data from adolescents or young adults. Specific strength standards for all young athletes (<16 years.) are lacking [58]. Therefore, the younger athletes were also administered the same strength levels 1–5.

### 2.3. Statistical Analysis

The data were analyzed using SPSS 26.0 (IBM, Ehningen, DE, Germany). In addition, analyses were conducted in R [59], Spearman’s Rho was calculated using the cor.test function, and their respective confidence intervals with the package spearman.ci. Figures were produced using the package ggplot2 [60]. The significance level for all statistical tests was set at < 0.05.

The best performance for each test was used for the statistical analysis. Descriptive statistics for all measures are presented as mean ± standard deviation (SD). The Kolmogorov–Smirnoff test for normality was calculated for the total data and the sub-groups’ data. The calculation by the Kolmogorov–Smirnoff test showed that, for the total group, SJ, CMJ, and REL SQ were normally distributed, and that 30 m LS data was not normally distributed. For subgroups (based on strength level), all variables were normally distributed, except LS and REL SQ for subgroups strength levels 2, 3, and 4. Since not all variables were normally distributed, bivariate one-tailed Spearman correlation analysis (Spearman rho) was used to assess the relationship between REL SQ with sprint and jump performance, respectively, for the total group and the subgroups. In addition, the partial correlation was calculated to control for a possible influence of the subjects’ age.

## 3. Results

The descriptive statistics (mean standard deviation) of the anthropometric and performance data are presented in Table 1 and Table 2, respectively. The athletes with strength levels 3 and 4 are, on average, older, taller and heavier than athletes with a lower strength level.

Athletes with higher strength levels exhibited higher mean values in jumping and sprinting performance. No soccer player achieved a maximum relative strength output above twice his body weight.

**Figure 1:**
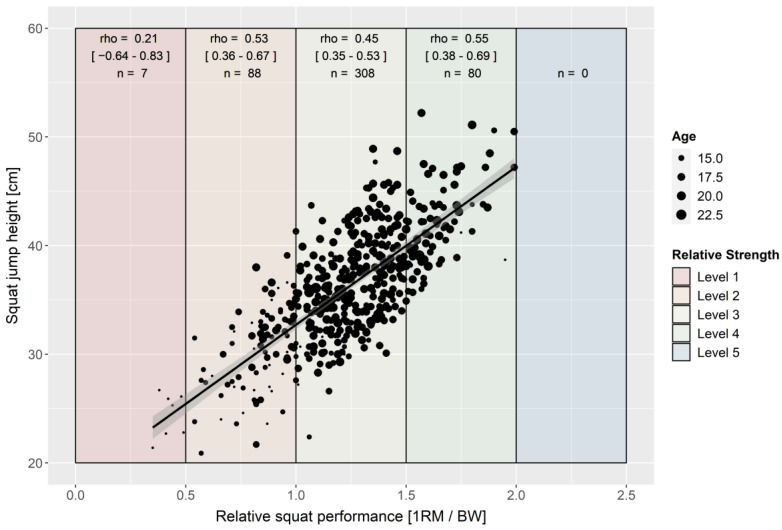
Scatterplot for relative squat and squat jump performance with trendline and 95% confidence interval (rho = 0.73 [*p* < 0.001; 95% CI = 0.69–0.77]; r_partial_ = 0.70 [*p* < 0.001; CI95% = 0.66–0.75]; *n* = 483).

**Figure 2:**
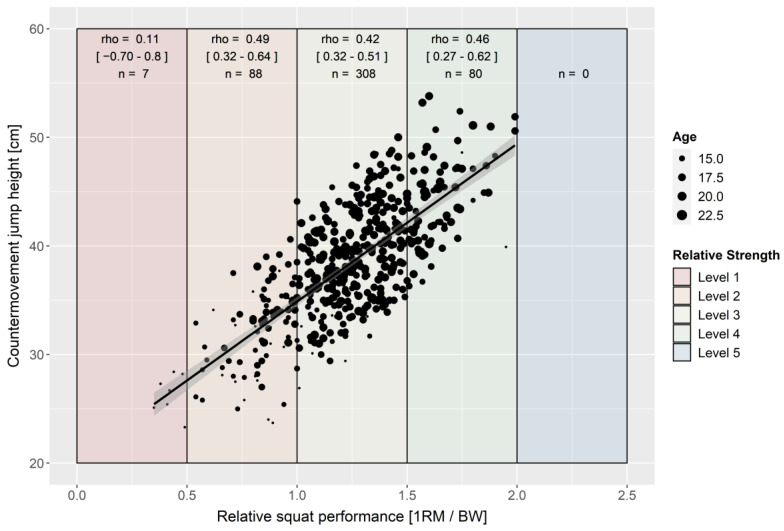
Scatterplot for relative squat and countermovement jump performance with trendline and 95% confidence interval (rho = 0.72 [*p* < 0.001; 95% CI = 0.67–0.76]; r_partial_ = 0.69 [CI95% = 0.64–0.73]; *n* = 483).

**Figure 3:**
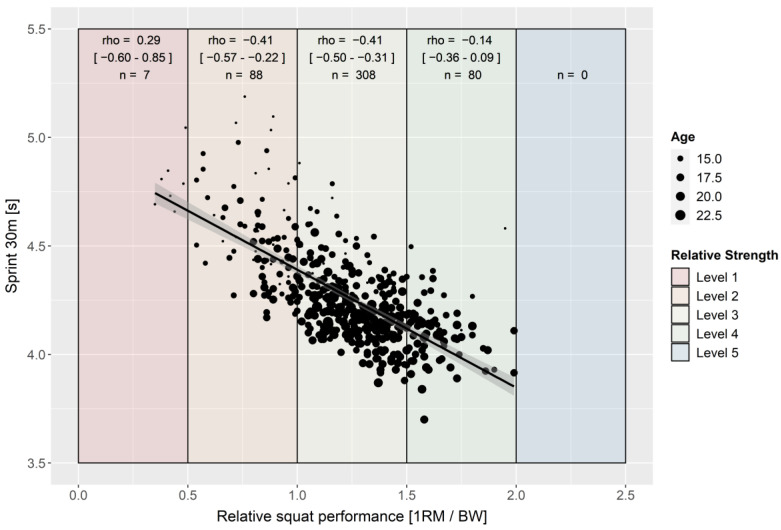
Scatterplot for relative squat and linear sprint performance with trendline and 95% confidence interval (rho = −0.67 [*p* < 0.001; 95% CI = −0.62–−0.72]; r_partial_ = −0.60 [CI95% = −0.54–−0.6]; *n* = 470).

Over the total group of soccer players, REL SQ correlates with all three performance parameters (rho = |0.67–0.73|, Figure 1, Figure 2 and Figure 3). REL SQ explains 45–53% of the variances of the tested jumping and sprinting performances. The correlation analysis of the various strength level subgroups revealed lower to no correlations within the subgroups (Figure 1, Figure 2 and Figure 3). In particular, the strength level 1 subgroup showed no significant correlation between REL SQ and any of the performance parameters (Figure 1, Figure 2 and Figure 3). Controlling for a possible age effect by partial correlation modifies the coefficients only marginally.

## 4. Discussion

This study was designed to examine the influence of REL SQ on jumping and sprinting performance in a large elite youth soccer sample. The results of this study show positive correlations of REL SQ with both jumping tasks (SJ and CMJ), and a negative correlation with the sprinting task (LS30). Overall, REL SQ explained 45–53% (r = |0.67–0.73|) of the variance of SJ, CMJ, and LS. Strength levels 2–4 showed similar coefficients of correlation with jumping performance (r = |0.42–0.55|) and levels 2–3 with sprint performance (r = |0.41|). The respective extreme strength levels showed lower correlations with jumping and sprinting performance (r = |0.11–0.29|). No coefficients of correlation could be calculated for strength level 5 because no subject achieved an appropriate strength level (>2.0 REL SQ).

The correlation coefficients of this study (r = |0.67–0.73|) are found to be approximately in the middle of the range of correlations found in the literature for free-weight squats (r = |0.28–0.94|) [30,31,32,34,36]. In principle, this does not seem surprising, since the coefficients of the cited studies are based on smaller samples (*n* = 15–34). The sampling error in these studies can, therefore, be assumed to be higher compared to this study. Consequently, a higher dispersion around the true coefficient can be expected in these studies, and it seems stringent to locate (roughly in the middle of the range of coefficients documented in the literature) the coefficients measured in this study, which should scatter less (due to the relatively large sample) around the true coefficient. Furthermore, the explained variance (45–53%) shows that, in addition to the influence of maximum strength, other variables (e.g., technique) can be expected to influence performance. Nevertheless, the data obtained in this study strengthen Schmidtbleicher’s theory [29] that maximum strength, as a basic strength ability, positively influences the performance of high-speed strength. Altmann et al. [61] and Pedersen et al. [41] were able to demonstrate that performances from test batteries (CMJ and LS) show moderate to strong (r = |0.37–0.86|) correlations with game performances, such as top speed or aerial duels won, among others. Consequently, a high maximum strength level can be assumed to be a performance determinant in the sport of soccer. Surprisingly, however, none of the evaluated strength performances could be classified in strength level 5. Strength level 5 (>2.0 REL SQ) is categorized as a good strength level in other sports [50,51,52,53,54,55,56,57] and can be well achieved through long-term planned strength training in team sports, such as soccer [44,62]. This raises the question of whether strength levels higher than twice the body weight do not increase players’ game performance further, or whether there is a potential performance gap that could be addressed with specific maximum strength training.

Suchomel’s theory, that the relationship between REL SQ and speed–strength capability is not solely linear but rather S-shaped [44], can neither be confirmed nor disproved by the data of this study. The trend line of the scatterplots (Figure 1, Figure 2 and Figure 3) shows that the confidence intervals are hyperbolic. In line with Suchomel’s theory [44], the further the values move away from the mean, the wider the confidence interval becomes. In general, when considering the subgroups (strength level), lower coefficients of correlation are observed compared to the total group. However, this can be explained mathematically by the clustering based on one variable. The intermediate strength level (SJ/CMJ: 2–4, LS 2–3) showed comparable correlation coefficients while the extreme levels showed lower correlations with the performance variables. This may indicate that REL SQ and speed-strength ability are S-shaped. Understandably, the lower coefficients in the subgroup with strength level 1 may be limited by the technical performances of the individuals and, consequently, underestimate their actual strength performance [44]. This is also supported by the fact that the youngest soccer players with the least training experience are found within strength level 1. It is possible that the S-shaped relationship depends not only on strength output, but also on the complexity of the target movement (comparison of the correlation coefficients [jump vs. sprint of the strength levels 4]). The higher the complexity of a movement is (jump vs. sprint performance), the more difficult the transfer of strength, which can probably be argued to reflect the task-specificity of the central nervous system [63]. Again, it should be noted that the effect may not be found in the data because no soccer player showed strength level 5 strength values (>2.0 REL SQ). Refuting this, it should be noted that the correlation coefficients of the extreme strength levels are based on lower sample sizes. This could lead to a relatively higher sampling error for these subgroups.

The limitations of this study concern the partly low/non-existent number of subjects with extreme strength levels. The calculated correlation coefficients of these strength levels should therefore be interpreted with caution. However, these distributions can be explained with the normal distribution of performances, while also emphasizing the need for future studies with very strong subjects (>2.0 REL SQ) [44]. An additional limitation is the evaluated age range. Possible age effects were controlled by partial correlation. Controlling for age via the partial correlation calculation shows only marginal changes in the coefficient of the Spearman calculation, which highlights that the observed correlations can be generalized across various age groups. This sensitivity test (comparison between Spearman and partial correlation) reinforces the fundamental influence of REL SQ on sprint and jump performance. Few subjects missed one of the respective measurements due to organizational reasons (jumping performance: *n* = 7; sprinting performance: *n* = 22). This should have had no/marginal effect on the results, considering the total sample size. Since not all variables/subgroups had a normal distribution, Spearman’s nonparametric coefficient was calculated for all correlations because of comparability. This may mean an underestimation of the real correlation coefficients for the normally distributed variables. After controlling, the non-parametric coefficients differ only marginally from the calculation with a parametric alternative. Despite these limitations, the results seem to strengthen the proposed relationships between squatting strength and various factors that influence players’ game performances.

## 5. Conclusions

REL SQ may prove useful as part of regular test batteries to monitor the performances of soccer players. The data from this study show a clear influence of REL SQ on sprint and jump performance. A basic recommendation to increase strength level for soccer players with a lower strength level can therefore be made [44]. Longitudinal studies have already shown that strength training is preventive, safe, and performance-enhancing [53,64,65]. From this, it can be concluded that long-term strength training is recommended for elite youth soccer players to increase their performance in sprinting and jumping, and thereby, to potentially increase their game performances. However, achieving a high level of strength should follow an optimal trend. Likewise, further studies are needed to evaluate the optimal strength range for elite youth soccer players.

## Figures and Tables

**Table 1:** Means and standard deviations of age and anthropometric data.

Group	*n*	Age (yrs.)	Body Mass (kg)	Height (m)
Level 1	7	13.0 ± 0.0	41.9 ± 3.7	1.53 ± 0.05
Level 2	89	14.0 ± 1.5	61.9 ± 14.9	1.71 ± 0.13
Level 3	317	16.8 ± 1.6	70.7 ± 9.7	1.78 ± 0.08
Level 4	77	17.1 ± 1.6	66.7 ± 8.1	1.77 ± 0.07
Level 5	-	-	-	-
Total	492	16.5 ± 1.8	68.07 ± 11.5	1.76 ± 0.10

yrs. = years old, kg = kilogram, m = meter, Level = strength level.

**Table 2:** Means and standard deviations of performance variables.

Group	Squat Jump (cm)	Countermovement Jump (cm)	30 m Linear Sprint (s)	Relative Strength Performance	Maximum Strength Performance (kg)
Level 1	24.4 ± 2.1	26.3 ± 1.8	4.80 ± 0.13	0.43 ± 0.05	17.6 ± 2.7
Level 2	30.5 ± 3.8	33.1 ± 3.7	4.52 ± 0.22	0.84 ± 0.12	52.5 ± 15.6
Level 3	36.4 ± 4.3	38.7 ± 4.3	4.22 ± 0.16	1.26 ± 0.13	88.9 ± 15.2
Level 4	42.2 ± 3.8	44.0 ± 4.1	4.10 ± 0.14	1.65 ± 0.12	109.8 ± 13.5
Level 5	-	-	-	-	-
Total	36.1 ± 5.5	38.2 ± 5.6	4.27 ± 0.22	1.23 ± 0.29	84.4 ± 24.3

cm = centimeters, s = seconds, kg = kilogram, Level = strength level.

## Data Availability

The data is not yet publicly available.
